# Association between physical activity and academic performance in Korean adolescent students

**DOI:** 10.1186/1471-2458-12-258

**Published:** 2012-04-02

**Authors:** Wi-Young So

**Affiliations:** 1 Department of Human Movement Science, Seoul Women’s University, Seoul, South Korea

## Abstract

**Background:**

Recently, physical activity (PA) was found to improve cognitive and memory functions in the brain; however, no epidemiological studies have specifically investigated this phenomenon in the Korean adolescent student population. The purpose of this study was to investigate the effects of various types of PA undertaken at various frequencies, on the academic performance of Korean adolescent students.

**Methods:**

A total of 75,066 adolescent students (39,612 males and 35,454 females) from the 7^th^ to the 12^th^ grades took part in the 5^th^ Korea Youth Risk Behavior Web-based Survey (KYRBWS-V) project, conducted in 2009. Using data acquired by that survey, potential relations between PA and academic performance were explored in this current study through multivariate logistic regression analysis incorporating adjustment for covariate variables including age, body mass index, the parents’ education level, and the income status of the family.

**Results:**

Compared with boys who did not regularly participate in any vigorous PA, those who did so 2, 3, or 4 times a week had greater odds of reporting an average or above-average academic performance. Compared with boys who did not participate in any moderate PA, those who did so 1, 2, 3, 4, or ≥5 times a week also had greater odds of reporting an average or above-average academic performance. Interestingly, when compared with boys who did not participate in any strengthening exercises, those undertaking strengthening exercises ≥5 times a week had lesser odds of reporting a below-average academic performance. Compared with girls who did not regularly participate in any vigorous PA, those who did so ≥5 times a week had greater odds of reporting an average or above-average academic performance. Compared with girls who did not participate in any moderate PA, those that did so 2 or 3 times a week had greater odds of reporting an average or above-average academic performance. Interestingly, when compared with girls who did not regularly participate in any strengthening exercises, those undertaking strengthening exercises ≥5 times a week had lesser odds of reporting a below-average academic performance.

**Conclusions:**

Our analyses of the relevant data from the KYRBWS-V suggested that vigorous PA was positively correlated with academic performance in the case of boys, and moderate PA was positively correlated with academic performance in both boys and girls. However, strengthening exercises were not positively correlated with academic performance in boys or girls. Furthermore, when undertaken 5 or more times a week, vigorous PA in boys and strengthening exercises in both boys and girls were negatively correlated with academic performance. The results from this study are potentially relevant to the development of future education policies in Korean schools, particularly with regard to early intervention strategies designed to identify and counteract potential factors contributing to academic underachievement.

## Background

Physical activity (PA) and exercise enhance the functioning of the musculoskeletal and cardiovascular systems [1,2]. Additionally, PA is known to confer benefits such as improvement in weight control, bone and muscle strength, mental health and mood, ability to perform daily activities, and life span as well as reduction in the risk of cardiovascular disease, type 2 diabetes, metabolic syndrome, certain cancers, and falls [3]. Furthermore, physical inactivity leads to a sedentary lifestyle that in turn may lead to overweight or obesity [4-6], and is known to have adverse health effects such as increased risk of cardiac disease, musculoskeletal disorders, stroke, type II diabetes, and certain cancers [7]. Therefore, adequate PA is considered important for good health.

Adolescence is an important period for establishing healthy habits [8]. For example, approximately 80% of obese adolescents grow into obese adults [9]. Many studies have investigated the relation between PA and health effects. The results of these studies suggest that it is important to increase PA and decrease sedentary activities to improve health [10,11]. Further, PA was recently found to improve cognitive and memory functions [12,13]. These reports showed that regular PA might improve the academic performance of adolescent students. In addition, several studies have reported that PA enhances academic performance and outcomes [14-17]. However, while studies conducted in various regions of the world suggest that PA has beneficial effects on academic performance, and some practical meta-analysis-based evidence associates PA with academic performance [18], there is no epidemiological evidence that indicates whether there is an association between PA and the academic performance of students in Korea. Furthermore, while some regional studies have analyzed this relation in Korean adolescents, no nationwide study has been conducted in this regard. Therefore, this study was conducted to investigate the relation between PA and academic performance in Korean adolescent students.

## Methods

### Subjects

The 5^th^ Korea Youth Risk Behavior Web-based Survey (KYRBWS-V), which was administered in 2009, was a retrospective epidemiological study with a complex sample design that included multistage sampling, stratification, and clustering. This was a national school-based survey conducted by the Korea Centers for Disease Control and Prevention (KCDCP) to estimate the prevalence of health-risk behaviors among adolescent Korean students from the 7^th^ to 12^th^ grade [19]. The KYRBWS-V used a 16-city cluster sample strategy, and the sampling frame of the survey included the entire country. A total of 400 middle and 400 high schools were sampled to evaluate the association between PA and the academic performance of adolescent Korean students, taking into account potential covariate variables. All the details of the data collection procedure have been reported by the KCDCP [19], and this survey has been reported to be valid and reliable [20,21]. The survey was administered to a nationally representative group of Korean adolescent students. Because the survey did not collect identifier information, ethical approval was not required. Further, students were given the option to not participate. The students who chose to participate in the study completed the questionnaire anonymously through the Internet, at their school. Teachers at participating schools were supplied with unique sets of ID numbers printed on tags, and each participating student drew one of these at random before accessing the survey web page and logging in using that unique ID number. A total of 75,066 adolescent students (39,612 boys and 35,454 girls) participated in the study, and the response rate was 97.6% (out of 76,937 students, 75,066 accessed the questionnaire online). The characteristics of the subjects are shown in Table 1.

**Table 1:** The characteristics of the subjects

**Variables**		**Boys** **(n = 39,612)**	**Girls** **(n = 35,454)**	**Total** **(n = 75,066)**
Age (years)		15.00 ± 1.73	15.12 ± 1.77	15.06 ± 1.75
Height (cm)		169.58 ± 8.19	160.08 ± 5.39	165.09 ± 8.46
Weight (kg)		60.14 ± 11.72	51.47 ± 7.67	56.04 ± 10.91
Body mass index (kg/m^2^)		20.80 ± 3.21	20.05 ± 2.58	20.45 ± 2.95
Family income status	Very rich	2,923 (7.4)	1,434 (4.0)	4,357 (5.8)
	Rich	8,985 (22.7)	6,908 (19.6)	15,893 (21.2)
	Average	17,743 (44.7)	17,706 (49.9)	35,449 (47.2)
	Poor	7,150 (18.1)	7,090 (20.0)	14,240 (19.0)
	Very poor	2,811 (7.1)	2,316 (6.5)	5,127 (6.8)
Father’s education level	Unknown*	7,297 (18.4)	5,321 (15.0)	12,618 (16.8)
	Middle school or lower	2,707 (6.8)	2,483 (7.0)	5,190 (6.9)
	High school	14,294 (36.1)	13,915 (39.2)	28,209 (37.6)
	College or higher	15,314 (38.7)	13,735 (38.8)	29,049 (38.7)
Mother’s education level	Unknown*	8,069 (20.4)	5,059 (14.3)	13,128 (17.5)
	Middle school or lower	2,447 (6.2)	2,490 (7.0)	4,937 (6.6)
	High school	17,745 (44.7)	18,212 (51.4)	35,957 (47.9)
	College or higher	11,351 (28.7)	9,693 (27.3)	21,044 (28.0)
Academic performance	Very good	04,958 (12.5)	03,454 (09.7)	08412 (11.2)
	Good	09,172 (23.2)	08,411 (23.7)	17583 (23.4)
	Average	10,544 (26.6)	09,675 (27.3)	20219 (26.9)
	Bad	09,661 (24.4)	09,477 (26.7)	19138 (25.5)
	Very bad	05,277 (13.3)	04,437 (12.5)	09714 (12.9)
Grade	7^th^	6,933 (17.5)	5,781 (16.3)	12,714 (16.9)
	8^th^	6,965 (17.6)	5,903 (16.6)	12,868 (17.1)
	9^th^	7,035 (17.7)	5,792 (16.3)	12,827 (17.1)
	10^th^	6,890 (17.4)	5,587 (15.8)	12,477 (16.6)
	11^th^	6,104 (15.4)	6,323 (17.9)	12,427 (16.6)
	12^th^	5,685 (14.4)	6,068 (17.1)	11,753 (15.7)

Data are expressed as mean ± SD or N (%).

* These records were omitted from the multivariate logistic regression analysis in Table 2.

### Independent variables

 Frequency of vigorous PA: The examples included digging, soccer, basketball, aerobics, heavy weight lifting, fast cycling, and swimming. The response options were classified into 6 groups: (1) no vigorous PA, (2) once a week, (3) twice a week, (4) thrice a week, (5) 4 times a week, and (6) 5 or more times a week.

 Frequency of moderate PA: The examples included cycling at a regular pace, carrying light loads, and playing table tennis, badminton, and doubles tennis. The response options were classified into 6 groups: (1) no moderate PA, (2) once a week, (3) twice a week, (4) thrice a week, (5) 4 times a week, and (6) 5 or more times a week.

 Frequency of strengthening exercises: The examples included sit-ups, push-ups, weight lifting, and weight training. The response options were classified into 6 groups: (1) no strengthening exercises, (2) once a week, (3) twice a week, (4) thrice a week, (5) 4 times a week, and (6) 5 or more times a week.

### Dependent variables

Academic performance was evaluated using the following question: *In the past 12 months, how has your average academic performance been?* The response options were classified into 5 groups: (1) very good performance, (2) good performance, (3) average performance, (4) bad performance, and (5) very bad performance. This variable included only the assessment of the academic performance of students according to examination scores, not taking into account their behavioral conduct, attendance, or other achievements. On the basis of their responses, the participants were divided into 2 groups: (1) those who reported below-average academic performance and (2) those who reported average or above-average academic performance.

### Covariate variables

Age: The age of the students defined by the KYRBWS-V data was used without any modification.

Body mass index (BMI): The students were asked to report their height and weight, and their BMI (kg/m^2^) was calculated from the data supplied by each student.

Father’s education level: The 4 response options were (1) unknown, (2) middle school or lower, (3) high school, and (4) college or higher. Data from students reporting “Unknown” were omitted from the multivariate logistic regression analysis.

Mother’s education level: The 4 response options were (1) unknown, (2) middle school or lower, (3) high school, and (4) college or higher. Data from students reporting “Unknown” were omitted from the multivariate logistic regression analysis.

Family income status: The 5 response options were (1) very rich, (2) rich, (3) average, (4) poor, and (5) very poor.

### Data analysis

All results from this study are shown as mean ± standard deviation (SD) values. The statistical software SPSS Complex Sample^TM^ version 18.0 (SPSS, Chicago, IL, USA) was used in all the analyses to evaluate the complex sample survey. For statistical assessments, we took into consideration the complex sampling design of the KYRBWS-V. To assess the associations between academic performance and PA, after adjusting for covariate variables such as age, BMI, parents’ education level, and family income status, multivariate logistic regression analyses were performed. Statistical significance was set at *p* < 0.05.

## Results

Multivariate logistic regression analysis of academic performance in relation to PA patterns, after adjusting for covariate variables including age, BMI, parents’ educational level, and family income status, is shown in Table 2.

**Table 2:** The multivariate logistic regression analysis for school records in relation to the PA patterns

		**Case**	**OR**	**95% CI**	**p**
Boys		39,612			
Academic performance ≥ Average (yes/no)	No vigorous PA	7,314	Reference		
	Once a week	7,067	1.029	0.925–1.145	0.602
	Twice a week	7,847	1.198	1.080–1.329	0.001**
	Thrice a week	6,994	1.287	1.156–1.433	<0.001***
	4 times a week	3,066	1.265	1.117–1.433	<0.001***
	5 times a week or more	7,324	0.886	0.801–0.981	0.020*
	No moderate PA	7,946	Reference		
	Once a week	8,379	1.121	1.021–1.232	0.017*
	Twice a week	8,351	1.337	1.216–1.471	<0.001***
	Thrice a week	6,401	1.460	1.315–1.621	<0.001***
	4 times a week	2,539	1.363	1.183–1.570	<0.001***
	5 times a week or more	5,996	1.135	1.013–1.271	0.029*
	No strengthening exercises	13,290	Reference		
	Once a week	8,413	0.968	0.894–1.048	0.423
	Twice a week	6,354	1.076	0.978–1.183	0.132
	Thrice a week	4,804	1.021	0.920–1.132	0.700
	4 times a week	1,966	0.910	0.785–1.054	0.209
	5 times a week or more	4,785	0.828	0.754–0.910	<0.001***
Girls		35,454			
Academic performance ≥ Average (yes/no)	No vigorous PA	15,676	Reference		
	Once a week	7,540	1.074	0.988–1.168	0.094
	Twice a week	5,628	0.958	0.869–1.057	0.396
	Thrice a week	3,661	0.943	0.832–1.069	0.361
	4 times a week	1,009	1.073	0.879–1.309	0.488
	5 times a week or more	1,940	1.532	1.336–1.757	<0.001***
	No moderate PA	13,059	Reference		
	Once a week	8,640	1.034	0.947-1.129	0.455
	Twice a week	6,471	1.113	1.008–1.229	0.035*
	Thrice a week	4,032	1.306	1.161–1.468	<0.001***
	4 times a week	1,164	1.065	0.893–1.270	0.481
	5 times a week or more	2,088	0.904	0.797–1.024	0.111
	No strengthening exercises	21,540	Reference		
	Once a week	7,045	0.995	0.922–1.075	0.906
	Twice a week	3,286	0.928	0.834–1.034	0.176
	Thrice a week	1,898	0.997	0.867–1.147	0.969
	4 times a week	564	0.764	0.579–1.009	0.058
	5 times a week or more	1,121	0.739	0.618–0.883	0.001**

OR, Odd Ratio; CI, Confidence Interval; PA, Physical Activity.

*p < 0.05 **p < 0.01 ***p < 0.001 tested by multivariate logistic regression analysis (adjusted for age, body mass index, parents’ education level, family income status).

Compared with boys who did not engage in vigorous PA, the following participants had greater odds of reporting an average or above-average academic performance: those who participated in vigorous PA twice a week (OR = 1.198, 95% CI = 1.080–1.329, *p =* 0.001), thrice a week (OR = 1.287, 95% CI = 1.156–1.433, *p* < 0.001), 4 times a week (OR = 1.265, 95% CI = 1.117–1.433, *p* < 0.001), and 5 or more times a week (OR = 0.886, 95% CI = 0.801–0.981, *p* = 0.020). Compared with boys who did not engage in moderate PA, the following participants had greater odds of reporting an average or above-average academic performance: those who participated in moderate PA once a week (OR = 1.121, 95% CI = 1.021–1.232, *p* = 0.017), twice a week (OR = 1.337, 95% CI = 1.216–1.471, *p* < 0.001), thrice a week (OR = 1.460, 95% CI = 1.315–1.621, *p* < 0.001), 4 times a week (OR = 1.363, 95% CI = 1.183–1.570, *p* < 0.001), and 5 or more times a week (OR = 1.135, 95% CI = 1.013–1.271, *p* = 0.029). Compared with boys who did not engage in strengthening exercises, those who participated in strengthening exercises 5 or more times a week had lesser odds of reporting an average or above-average academic performance (OR = 0.828, 95% CI = 0.754–0.910, *p* < 0.001).

Compared with girls who did not engage in vigorous PA, those who participated in vigorous PA 5 or more times a week had greater odds of reporting an average or above-average academic performance (OR = 1.532, 95% CI = 1.336–1.757, *p* < 0.001). Compared with girls who did not engage in moderate PA, the following participants had greater odds of reporting an average or above-average academic performance: those who participated in moderate PA twice a week (OR = 1.113, 95% CI = 1.008–1.229, *p* = 0.035) and thrice a week (OR = 1.306, 95% CI = 1.161–1.468, *p* < 0.001). Compared with girls who did not engage in strengthening exercises, those who participated in strengthening exercises 5 or more times a week had lesser odds of reporting an average or above-average academic performance (OR = 0.739, 95% CI = 0.618–0.883, *p* = 0.001).

## Discussion

The aim of this study was to investigate the relation between PA and academic performance in adolescent Korean students. The results showed that vigorous PA was positively correlated with academic performance in the case of boys, and moderate PA was positively correlated with academic performance in both boys and girls. However, strengthening exercises were not positively correlated with academic performance in boys or girls. Furthermore, PA and strengthening exercises—when performed 4 times a week or more—were negatively correlated with academic performance in both boys and girls.

Three hypotheses have been proposed to explain the effect of PA or exercise on the brain. First, PA and exercise increase oxygen saturation and angiogenesis in the regions of the brain essential for performing tasks [22,23]. Second, they also increase the activity of neurotransmitters such as serotonin and norepinephrine in the brain, facilitating information processing [24-26]. Third, PA and exercise upregulate neurotrophins such as brain-derived neurotrophic factor, insulin-like growth factor-I, and basic fibroblast growth factor. These neurotrophins support neuronal survival and differentiation in the developing brain [27].

The results of our epidemiological study indicate that PA may increase brain and memory function and that moderate PA has a positive effect on the academic performance of both boys and girls. However, vigorous PA has a positive effect on academic performance only in the case of boys. This difference might be due to the fact that boys undergo rapid physical growth during adolescence [28] and generally prefer sports-related activities such as soccer and basketball, which are defined as vigorous PA according to the KYRBWS-V. In contrast, girls prefer leisure activities that are considered moderate PA [29]. For this reason, we believe that vigorous PA has a positive effect on the academic performance of boys.

Strengthening exercises were not positively correlated with academic performance in the case of boys or girls. PA usually affects the circulatory system [1]. Thus, PA stimulates the brain by increasing blood circulation to the entire body. However, strengthening exercises focus on the musculoskeletal system and not on the circulatory system [2]; therefore, because strengthening exercises stimulate the muscles, they may stimulate brain functions only slightly through increased blood circulation to the entire body. We also found that vigorous PA in boys and strengthening exercises in both boys and girls performed at a frequency of 5 or more times a week negatively affected academic performance. Performing vigorous PA and strengthening exercises 5 or more times a week is time consuming and would potentially deprive students of study time that could be invested in improving their academic performance.

This study has some limitations. First, it was conducted online; therefore, the family income status, parents’ educational qualifications, and the students’ height and weight were not directly measured; they were recorded by the students themselves. Therefore, these data might be inaccurate. Second, this study did not investigate whether PA might affect attention and alertness at school. Therefore, further studies will be required to determine the extent to which these variables might contribute to attention and alertness in students. Third, the academic performance variable did not incorporate behavioral conduct or attendance at school, and these factors might contribute to school performance. Fourth, this study was a retrospective cohort study; thus, we examined only the interrelation between variables, not cause and effect. However, the subject pool was extremely large and highly representative of the wider Korean population (75,066 subjects residing in Korea). Therefore, these results, derived from the KYRBWS-V, can be considered a valid indicator of the relation between PA and academic performance in Korean adolescent students.

## Conclusions

We conclude that vigorous PA undertaken less than 4 times a week was positively correlated with academic performance in the case of boys, and moderate PA was positively correlated with academic performance in both boys and girls, in the adolescent Korean population. However, strengthening exercises were not positively correlated with academic performance in boys or girls. Furthermore, when performed 5 or more times a week, vigorous PA in boys and strengthening exercises in both boys and girls were negatively correlated with academic performance.

Adequate PA can provide health benefits and play a vital role in weight control and reducing the risk of chronic diseases. Furthermore, this study shows that physically active adolescents are more likely to perform better at school. On the basis of the results of this study, we suggest that health-care providers incorporate PA in the students’ time tables and encourage them to be physically active and consequently improve academic performance.

## Competing interests

The author has no competing interests.

## Author’s contributions

W-YS contributed to study design and management, performed statistical analyses and drafted the manuscript.

## Pre-publication history

The pre-publication history for this paper can be accessed here:

http://www.biomedcentral.com/1471-2458/12/258/prepub
